# Insulin promotes progression of colon cancer by upregulation of ACAT1

**DOI:** 10.1186/s12944-018-0773-x

**Published:** 2018-05-24

**Authors:** Xin Chen, Huiling Liang, Qibin Song, Ximing Xu, Dedong Cao

**Affiliations:** Department of Oncology, Wuhan University, Renmin Hospital, Wuhan 430060, Hubei Province People’s Republic of China

**Keywords:** Insulin, Colon cancer, ACAT1

## Abstract

**Background:**

Insulin resistant and the progression of cancer is closely related. The aim of this study was to  investigate the effect of insulin on the proliferation and migration of colon cancer cells and its underlying mechanism.

**Methods:**

Colon carcinoma tissues from the 80 cases of colon cancer patients were collected. Immunohistochemistry was used to detect the expression of acyl coenzyme A: cholesterol acyltransferase1 (ACAT1), and we analyzed the correlation between hyperglycemia and ACAT1, hyperglycemia and metastasis. CCK8 assay and transwell assay were used to investigate the effect of different concentrations of insulin and ACAT1siRNA on human colon cancer cell line HT-29. ACAT1 mRNA expression and protein level in HT-29 cells were determined by real-time quantitative PCR and western blotting, respectively.

**Results:**

Biopsies from patients with colon carcinoma showed hyperglycemia links ACAT1, lymph nodes metastasis and distant metastasis. Insulin markedly promoted cell proliferation and migration in human colon cancer HT-29 cells. Moreover, ACAT1mRNA expression and protein level were increased by insulin. ACAT1siRNA resulted in a complete inhibition of the ACAT1 mRNA expression. Consequently insulin-triggered cell proliferation and migration on colon cancer cells were inhibited.

**Conclusion:**

The progression of colon cancer has a positive correlation with hyperinsulinemia. Insulin-triggered cell proliferation and metastatic effects on colorectal cancer cells are mediated by ACAT1. Therefore, insulin could promote colon cancer progression by upregulation of ACAT1, which maybe is a potential therapeutic target for colon cancer.

## Background

Colon cancer is one of the common clinical cancers. Facts such as diet and obesity have been shown to increase the risk of developing colon cancer [1]. Insulin resistance, a crucial mechanism that links obesity, diabetes mellitus type 2, and the metabolic syndrome. The current study found insulin resistant and the progression of cancer is closely related [2]. The pathophysiological mechanisms some are still to be discovered. Our previous research has considered that insulin unregulated the expression of ACAT1 in cultured macrophages [3, 4]. ACAT1 is an important enzyme in the pathways of cholesterol esterification. An important feature of tumor is a high content of cholesteryl esters stored in lipid droplets. Recent studies have focused to ACAT1 and its effects on cancer cell growth. However, few studies have been done on the mechanism of ACAT1 in insulin-related colon cancer. We aim to test the effect of insulin on proliferation and migration of colon cancer cells. Furthermore, we propose to test the feasibility of ACAT1 be involved in.

## Methods

### Chemical and reagents

All standard chemicals were purchased from commercial sources and were all of analysis grade, unless stated otherwise. ACAT1siRNA were from Qiagen (USA). Primers for real-time PCR kit were from Thermo Fisher Scientific (China). Rabbit polyclonal antibody against ACAT1 was bought from Cayman Chemical (USA). Penicillin/streptomycin, RPMI 1640 culture medium, fetal bovine serum (FBS), 0.25% trypsin digestion liquid, insulin and all other chemicals and reagents were purchased from Sigma-Aldrich (St. Louis, MO, USA).

### Patients and data collection

The clinical data of 80 patients with colon cancer were collected from March, 2016 to March, 2017 at Wuhan University Renmin hospital. The study was approved by the ethics committee at Wuhan University Renmin hospital and was conducted in accordance with the Declaration of Helsinki and Ethical Guidelines for Clinical Research. All patients provided written informed consent. The patients were classified by fasting insulin (FINs) (> = 85 pmol/L and < 85 pmol/L), and the associations between ACAT1 expression and clinical features were assessed by the chi-squared test. *P* value of < 0.05 was considered significant for all analysis.

ACAT1 analysis tissue was prepared and colon carcinoma was independent confirmed by two pathologists, ACAT1 analysis tissue was stained by immunohistochemistry (IHC) and IHC staining was carried out using image pro plus 6.0.

### Cell culture

HT-29 cells, a human colon adenocarcinoma cell line, were purchased from Wuhan University (Wuhan, China). HT-29 cells were maintained in McCoy’s 5A medium supplemented with 3 mmol/L L-glutamine, 10% (*v*/v) heat-inactivated bovine fetal serum, and a mixture of antibiotics to give a final concentration of 50 μg/ml penicillin, 50 μg/ml streptomycin, 50 μg/ml gentamicin, and 1.25 μg/ml amphotericin B, in a humidified atmosphere at37°C and 5% CO2. Culture medium was changed every 3 days. Before assay, cultured cells were harvested, washed once again with ice-cold PBS (calcium- and magnesium-free).

### RNA extraction and quantitative real-time PCR

Total cellular RNA was extracted from HT-29 cells using the high fidelity RT-PCR kit according to manufacturer’s instructions. Total RNA was reverse transcribed to generate cDNA for subsequent RT-PCR. Platinum SYBR Super mix was used to amplify sequences for ACAT1and the housekeeping gene GAPDH in HT29 cells. Reactions were performed using specific primers; using 5’-GCA GGCTTA CCTATT TCTACT C-3′, and 5’-C AGT TAG CCC GTCTTT TACAAT C-3′ for ACAT1; primers for GAPDH are 5’-GAACGG TGA AGG TGA CA-3′, and 5′-TAG AGA GAA GTGGGGTGG-3′. Real-time RT-PCR was carried out on the smart cycler as follows: incubate at 94 °C for 30 min and 1 cycle of 94 °C for 2 min hold (hot-start) followed by 35 cycles of 94 °C for 30s, annealing temperature at 57 °C for 30s and 72 °C for 30s. The number of PCR cycles to reach the fluorescence threshold was the cycle threshold (Ct). The Ct value for each sample was proportional to the log of the initial amount of input cDNA. In the present study, we used the 2-ΔΔCt methods to analyze the relative changes in gene expression from real-time quantitative PCR experiments. ΔΔCt = (Ct_target_ − Ct_GAPDH_)_Sample A_ − (Ct_target_ − Ct_GAPDH_)_Sample B_.

### Protein extraction and western blot analysis

After washing three times with ice cold PBS, cells were harvested in a lysis buffer. According to the published article [5], The protein samples were then subjected to 10% sodium dodecyl sulfate polyacrylamide gel electrophoresis and transferred to a nitrocellulose membrane (Bio-Rad, Hercules, CA, USA). The membranes were blocked with 5% skim milk in Tris-buffered saline containing Tween-20 (TBST) for 1 h, and were probed with primary antibodies overnight at 4 °C. After washing three times with 0.02 M Tris-buffered saline containing 0.05%Triton X-100 (pH 7.5), the membrane was incubated with horseradish peroxidase-conjugated anti-rabbit IgG antibody (goat anti-rabbit IgG, 1:5000 dilution) at 37 °C for 1 h. ACAT1 was visualized by a chemiluminescence’s method (ECL Western blotting detection system). The bands were quantitated using Bio-Rad’s Quantity One 1-D analysis software. The experiments were repeated at least thrice.

### Cell viability assay

Cell proliferation was assessed using a Cell Counting Kit-8 (CCK-8, Dojindo, Japan) according to the published article [6]. HT-29 cells in the logarithmic phase were harvested and resuspended at a final concentration of 5 × 10^4^cells/ml, then were seeded into 96-well tissue plates with 100 μl of cell solution each well in triplicate. Subsequent culturing for 24 h at 37 °C, the control group was added with an equal volume of culture medium and the cells in the experimental group were treated with 1, 10, 100 and 1000 nmol/L insulin, respectively. After incubating for an additional 12 h, 24 h, 48 h and 72 h, 10 μl CCK-8 solutions was added to each well, the plates were incubated at 37 °C for 4 h. Finally, Optical density (OD) value was measured at a wavelength of 450 nm using an ELISA reader. Each treatment was performed in triplicate.

### Transwell assay

Cell migration was assessed by using transwell chambers. HT-29 cells were resuspended with serum-free DMEM medium and adjusted the density to 2.5 × 105/ml. 200 μL cells seeded into the upper chambers, and the lower chambers were filled with DMEM-F12 medium containing 10% FBS as a chemoattractant. The plates were incubated in a 37 °C humidified incubator with 5% CO2 for 48 h, then membranes were removed, the cells on the upper surface of the chambers were removed gently and the migrated cells on the surface of the lower chambers were fixed in methanol, stained with crystal violet. The cells in 5 randomly-selected fields of view were imaged using a light microscope and the number of the cells were counted using ImageJ software. The experiments were performed in triplicate.

### Statistical analysis

Statistical analysis was carried out by GraphPad Prism 6.0 (La Jolla, CA, USA). Results presented are the mean ± SD from at least three independent experiments performed in duplicate. Statistical analysis was performed using chi-squared test or the two independent sample t-test. *P* value of < 0.05 was considered significant for all analysis.

## Results

### Hyperinsulinism was associated with ACAT1 expression and metastatic in colon cancer patients

Clinical features of colon cancer patients are summarized in Table 1. Of 80 colon cancer patients, 49 (61%) had hyperinsulinism (FINs> 85 pmol/L). ACAT1 expression, nodal status and metastatic status were analyzed in the overall population, demonstrating that ACAT1 expression (*P* <  0.05), nodal status (P <  0.05) and metastatic status (*P* <  0.01), were associated with an increased blood insulin levels.

**Table 1:** Clinical characteristic and ACAT1 expression of colon cancer patients - comparison between patients high FINs (> = 85 pmol/L) or low FINs (< 85 pmol/L)

Overall population		FINs(pmol/L)		*P* value
		> = 85	< 85	
		*N* = 49	*N* = 31	
Age (yr)	mean ± SD	59 ± 1.3	62 ± 2.1	> 0.05
Sex	males	28	17	> 0.05
	females	21	14	
ACAT1	positive	42	19	< 0.05
	negative	7	12	
Nodal status	positive	40	18	< 0.05
	negative	9	13	
Metastatic status	positive	20	3	< 0.01
	negative	29	28	

### Insulin promoted cell proliferation and migration of colon cancer HT29 cells

**Fig. 1:**
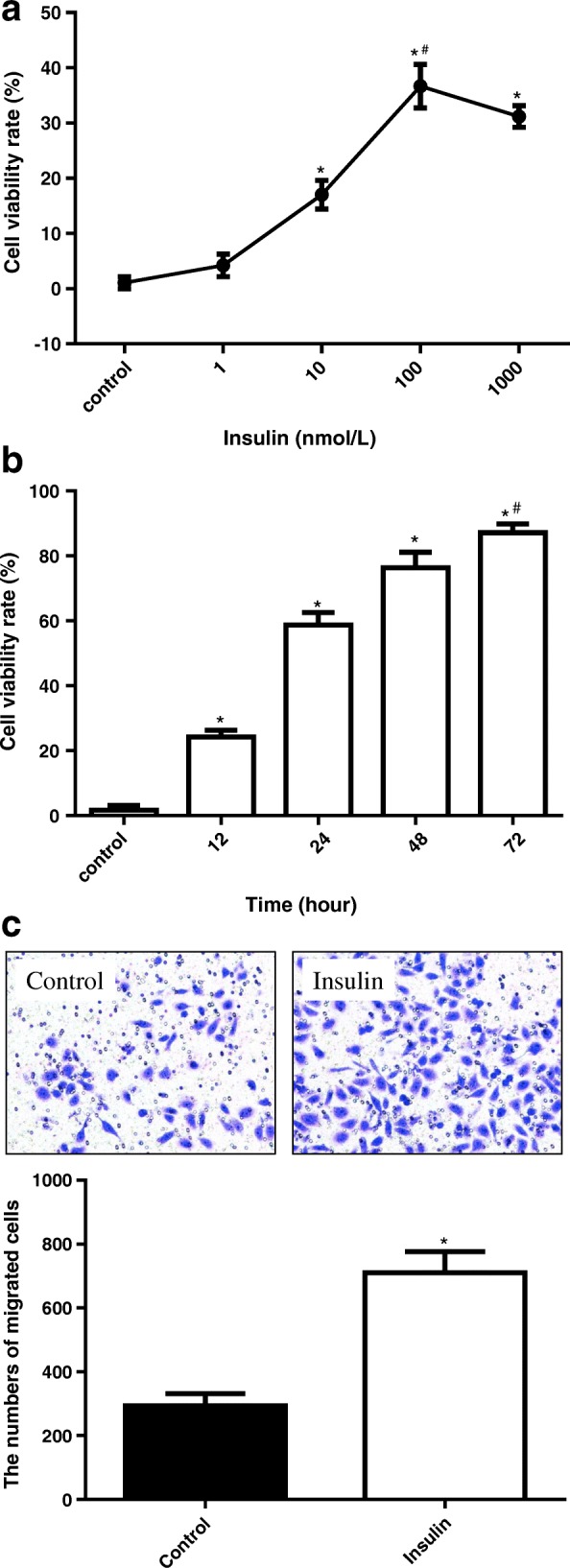
**a** The effects of different concentration insulin on the cell viability rate of HT-29 cells, PBS as control. Mean ± SEM, *n* = 5, 3 times. *P < 0.01 10 nmol/L group, 100 nmol/L group or 1000 nmol/L group vs. control group; #*P* < 0.01 1 nmol/L group or 10 nmol/L group vs. 100 nmol/L group. **b** The effects of insulin (100 nmol/L) on the cell viability rate of HT-29 cells at 0, 12, 24, 48 and 72 h, 0 h as control. Mean ± SEM, n = 5, 3 times. *P < 0.01 12 h group, 24 h group,48 h group, or 72 h group vs. Control group; #*P* < 0.01 12 h group, 24 h group or 48 h group vs. 72 h group. **c** The effect of insulin (100 nmol/L) on the migrative ability of HT-29 cells at 48 h, PBS as control. Mean ± SEM, n = 5, 3 times. *P < 0.01 insulin group vs. control group

To identify the effect of insulin on colon cancer cell growth, we tested the cell viability rate of the human colon cancer HT29 cells using CCK-8 assay in a 96-well format. HT29 cells were exposed to different concentration insulin. As shown in Fig. 1a and b, the results of CCK-8 assay indicated that insulin improved the viability of HT29 cells dose and time -dependently (*P* <  0.01). The effect on HT-29 cells started from concentrations as low as 10 nM, was noticeable at higher concentration (100 nM) at 48 h of treatment (*P* <  0.01). So we chose 100 nM and 48 h as the follow-up experiment condition.

To evaluate the effect of insulin on migration of colon cancer cells, transwell migration assays was applied in HT-29 cells which were treated with insulin (100 nM) for 48 h. The results showed that the number of migrated cells in insulin group was more than in PBS treated control group (Fig. 1c, *P* <  0.01).

### Insulin up-regulated the expression of ACAT1 gene and protein of HT-29 cells

**Fig. 2:**
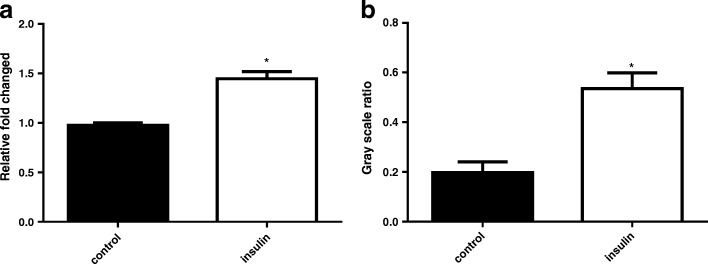
**a**-**b** The effects of insulin on the expression of ACAT1 gene and protein in HT-29 cells. **a**: ACAT1 mRNA was quantitated by SYBR Green I real time PCR (normalized to GAPDH), PBS as control. **b**: ACAT1 protein was quantitated by western blot (normalized to GAPDH), PBS as control. The data represent the mean±SD of three independent experiments. T-test was performed to determine statistical significance. * indicate differences of *P* < 0.01, compared with control group

To determine whether insulin may be involved in regulation of ACAT1; HT-29 cells were treated with insulin (100 nM) for 48 h. The data showed that insulin treatment resulted in up-regulation of the expressions of ACAT1mRNA and ACAT1 protein. The distinction of statistics is significant as compared with control group (Fig. 2a and b, *P* < 0.05).

### Effect of insulin on cell proliferation and migration of HT-29 cells was significantly blocked by ACAT1 siRNA

To substantiate the role of ACAT1 in colon cancer growth and metastasis promoted by insulin, we used ACAT1 siRNA to inhibit ACAT1 gene expression in HT-29 cells as described in Materials and Methods. To test the effect of ACAT1 siRNA on the proliferation and migration of HT-29 cell in vitro, we carried out CCK-8 assay and transwell assay.

**Fig. 3:**
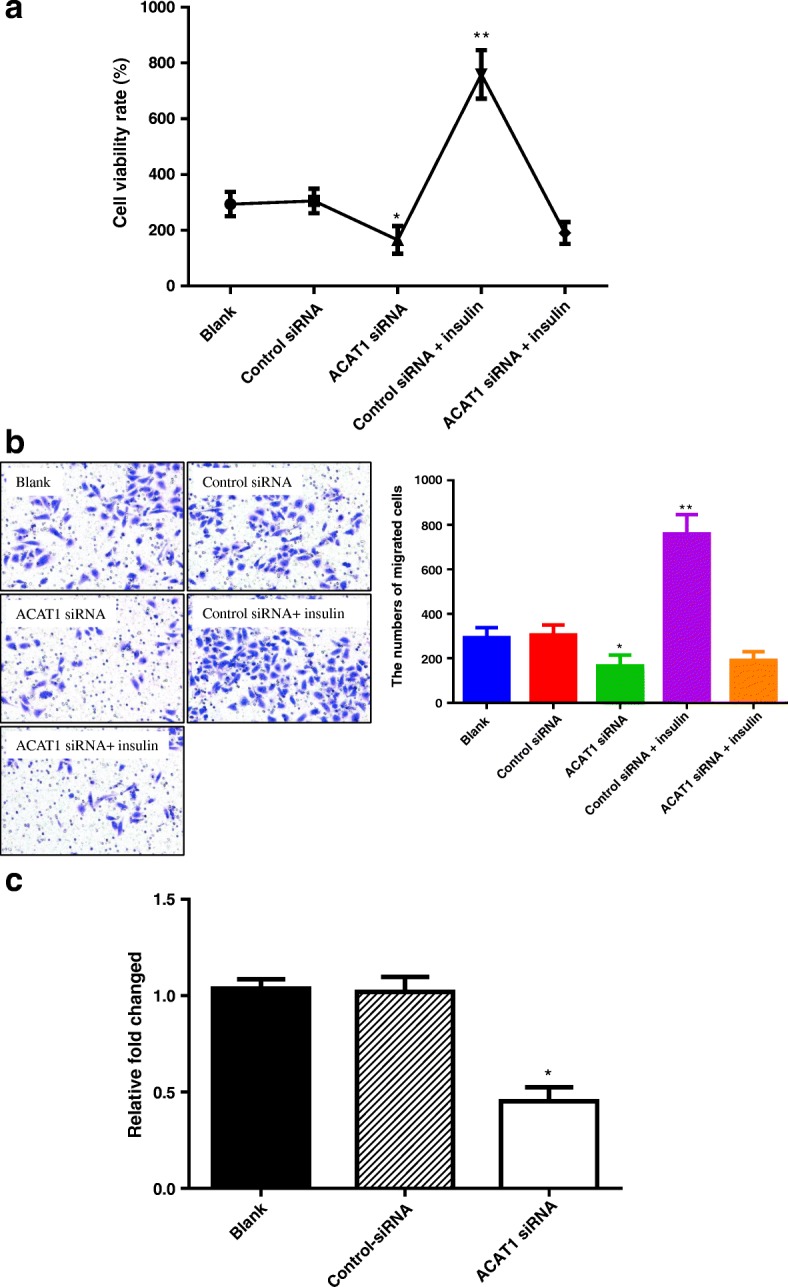
ACAT1 siRNA significantly blocked the role played by insulin in tumor growth and metastasis. **a**: the effect of ACAT1siRNA on the inhibition of HT-29 cells proliferation in vitro in a CCK-8 assay, PBS as Blank group, Control siRNA as Control siRNA group. Data are representative of three CCK-8 assays independently performed, *P < 0.01 ACAT1 siRNA group vs. Blank group or Control siRNA group, **P < 0.01 Control siRNA + insulin group vs. Control siRNA group or ACAT1 siRNA + insulin group; **b**: the effect of ACAT1siRNA on the inhibition of HT-29 cells migration in vitro in a chemotaxis assay. Data are representative of three chemotaxis assays independently performed, *P < 0.05 ACAT1 siRNA group vs. Blank group or Control siRNA group, **P < 0.01 Control siRNA + insulin group vs. Control siRNA group or ACAT1 siRNA + insulin group; **c**: Equal doses of ACAT1 siRNA and control siRNA were used to transfer HT29 cells as indicated to inhibit ACAT1 expression. The data are expressed as the relative fold change. Data shown represent the averages of three replicates for each group analyzed via the 2-∆∆Ct method. *P < 0.01 ACAT1 siRNA group vs. Blank group or Control- siRNA group

As shown in Fig. 3a, ACAT1 siRNA-treated HT-29 cells demonstrated significantly (*P* < 0.001) less proliferation ability than blank and negative-control. As shown in Fig. 3b, ACAT1 siRNA-treated HT-29 cells demonstrated significantly (*P* < 0.001) less migration ability than blank and negative-control, too. To confirm the efficacy of ACAT1 siRNA in ACAT1 gene silencing, equal doses of ACAT1 siRNA and negative-control siRNA were used to transfer HT-29 cells for 48 h. Fig. 3c shows that transfer of HT-29 cells for 48 h demonstrates significantly (*P* < 0.001) silenced ACAT1 gene.

## Discussion

In this research, we focused the relationship between insulin and colon cancer, and we found insulin-triggered cell proliferation and metastatic effects on colorectal cancer cells are mediated by ACAT1.

In worldwide, colon cancer is the third most commonly diagnosed cancer in men and the second in women [7]. Recent many studies have demonstrated the association between lifestyle and the development of colon cancer, including obesity, physical inactivity, and certain dietary factors [8], on the contrary, healthier lifestyle include maintaining a healthy body weight and being physically active was not only preventive method for colon cancer but also associated with decreasing colon cancer mortality rates [9].

In order to clarify the mechanism of unhealthy lifestyles lead to colon cancer, more and more studies found that lifestyle affect the regulation of insulin, and the insulin plays an important role in the initiation of cell growth and the proliferation of the colon cancer [10]. A lot of studies have observed increased levels of insulin have been related with an increased risk of colon cancer [11–13].

Meanwhile,it has been suggested that insulin may promote colorectal carcinogenesis directly by activating its own receptor, the receptors for IGF-I, or hybrid insulin/IGF-I receptors. These results indicate that insulin may play an important role in colorectal carcinogenesis [14].

When insulin binds to its receptor, PI3K pathways can be activated and cause cell proliferation and survival. Polymorphism of insulin gene and its association with colorectal cancer were demonstrated by some studies [11].

In our previous studies we found that high insulin increased ACAT mRNA and protein expression in macrophages, resulting in high amounts of cholesterol ester accumulation in cells, promoting foam cell formation, accelerate the development of atherosclerosis, which ERK/p38MAPK/JNK multiple signaling pathways involved [3, 4].

Cellular cholesterol is found in two forms: free cholesterol (FC) and cholesteryl esters (CE), ACAT uses cholesterol and long-chain fatty acyl coenzyme A as substrates to convert FC into CE. Cholesteryl ester hydrolases (CEH) are responsible for the reverse reaction converting CE into FC. ACAT and CEH act in opposite directions to maintain the dynamic equilibrium between FC and CE [15].ACAT is an enzyme in cells only known catalytic free cholesterol and long-chain fatty acids to form cholesterol esters, currently found in two forms ACAT isoenzymes, namely ACAT1 and ACAT2 [16]. ACAT1 distributed more widely, in macrophages, endothelial cells, the nerve cells are distributed, while ACAT2 mainly in the intestinal villus epithelial cells and liver cells [16]. ACAT not only closely related to atherosclerosis [17] and Alzheimer’s disease [18]. Even more studies found that ACAT / cholesterol esteris a new way to promote the occurrence and development of tumor cells [19]. Matsumoto et al. [20] identified strong expression of ACAT-1 in clear cell type renal cell carcinoma, and upregulation of ACAT-1 leads to high ACAT enzymatic activity, which accelerates the accumulation of cholesterol ester and is associated with tumor grade. Lee et al. [21] reported that ACAT-1 inhibitor significantly reduced cholesteryl ester storage in lipid droplets and elevated free cholesterol levels, which led to suppression of proliferation and apoptosis of colon cancer cell lines. But Uda [22] found that proliferation of CEM-CCRF cells was slightly affected by ACAT inhibitor; CE content in lipid droplets was significantly higher than those in control cells, and the enzyme activity was continuously inhibited. Antalis et al. [23] found that estrogen receptor negative (ER(−)) breast cancer cells had higher expression of ACAT1 as compared to ER(+)breast cancer cells, and proliferation of ER(−)breast cancer cells was reduced by inhibition of ACAT. Bemlih et al. [24] found that Avasimibe, a specific inhibitor of ACAT, inhibited the growth of the cells on glioma cell lines (U87, A172 and GL261). Paillasse et al. [25] found that inhibition of cholesteryl ester formation and ACAT activity by Sah58-035decreased by 34 and 73% (CCK2R-E151A) cell growth and invasion. Compared with normal cells, ACAT expression and activity were upregulated in many tumor cells, and the level of accumulation of cholesterol ester is also associated with the growth rate of tumor cells. Compelling evidence has implicated ACAT1 inhibitor in the inhibition of the growth and development of various tumor cells. ACAT1 inhibitor shows anti-neoplastic activity in a variety of experimental models in vivo and in vitro.

In mice treatment of ACAT-1 inhibitor notably suppressed tumor growth and extended the length of survival time [21]. De medina et al. [26] had same discovers that auraptene inhibits ACAT and binds to ERs correlated well with the control of growth and invasiveness of tumor cells in intact cancer cells of murine and human origins. ACAT/cholesterol esterification is a novel pathway that contributes to tumor cell proliferation and invasion.

On the other hand, data show that over expression of ACAT1/2 in human breast cancer cells using a lentiviral approach directly promotes tumor growth and lung metastasis [27].

In our previous studies we found that ACAT1 is overexpressed in colon cancer tissues compared with the paired adjacent normal tissues, which suggests that ACAT1 is involved in the pathogenesis of colon cancer.

In the present study, we found that blood insulin levels was associated with ACAT1 expression and clinic pathological characteristics, then we observed insulin improved cell proliferation and migration of colon cancer HT29 cells in a time and dose-dependent manner. In a follow-up experiment, we found that inhibition of ACAT1 prevents the insulin mediated HT29 cells cell proliferation and migration. In future studies, we will confirm our results in other colon cancer cell lines, and search for potential molecular targets of ACAT1 and identify them.

## Conclusion

In summary, we speculate that insulin could increase cellular proliferation and migration of the HT-29 cells by increasing the ACAT1 activity. These findings could explain why colon cancer cells proliferation and migration are particularly accelerated in insulin-resistant individuals and why colon cancer correlates closely with blood insulin levels, and this could lead to new treatment strategies.

## Abbreviations

CCK8: Cell Counting Kit-8
ACAT1: Acyl coenzyme A: cholesterol acyltransferase1
FINs: Fasting insulin
IHC: Immunohistochemistry
IR: Insulin resistant
SiRNA: Small interfering RNA
